# The therapeutic effect of a novel GAPDH inhibitor in mouse model of breast cancer and efficacy monitoring by molecular imaging

**DOI:** 10.1186/s12935-024-03361-x

**Published:** 2024-05-29

**Authors:** Yun-Qi Zhang, Wei Zhang, Xiang-Tai Kong, Wang-Xi Hai, Rui Guo, Min Zhang, Su-Lin Zhang, Biao Li

**Affiliations:** 1 Department of Nuclear Medicine, Ruijin Hospital, Shanghai Jiao Tong University School of Medicine, 197 Ruijin Second Road, Shanghai, 200025 China; 2 Collaborative Innovation Center for Molecular Imaging of Precision Medicine, Shanxi Medical University, Taiyuan, 030000 China; 3 Drug Discovery and Design Canter, State Key Laboratory of Drug Research, Shanghai Institute of Materia Medica, Chinese Academy of Sciences, 555 Zuchongzhi Road, Shanghai, 201203 China; 4 University of Chinese Academy of Sciences, No. 19A Yuquan Road, Beijing, 100049 China

**Keywords:** Glyceraldehyde-3-phosphate dehydrogenase, Inhibitor, Breast cancer, Warburg effect, Positron emission tomography/computed tomography

## Abstract

**Background:**

Breast cancer is a serious threat to women’s health with high morbidity and mortality. The development of more effective therapies for the treatment of breast cancer is strongly warranted. Growing evidence suggests that targeting glucose metabolism may be a promising cancer treatment strategy. We previously identified a new glyceraldehyde-3-phosphate dehydrogenase (GAPDH) inhibitor, DC-5163, which shows great potential in inhibiting tumor growth. Here, we evaluated the anticancer potential of DC-5163 in breast cancer cells.

**Methods:**

The effects of DC-5163 on breast cancer cells were investigated in vitro and in vivo. Seahorse, glucose uptake, lactate production, and cellular ATP content assays were performed to examine the impact of DC-5163 on cellular glycolysis. Cell viability, colony-forming ability, cell cycle, and apoptosis were assessed by CCK8 assay, colony formation assay, flow cytometry, and immunoblotting respectively. The anticancer activity of DC-5163 in vivo was evaluated in a mouse breast cancer xenograft model.

**Results:**

DC-5163 suppressed aerobic glycolysis and reduced energy supply of breast cancer cells, thereby inhibiting breast cancer cell growth, inducing cell cycle arrest in the G0/G1 phase, and increasing apoptosis. The therapeutic efficacy was assessed using a breast cancer xenograft mouse model. DC-5163 treatment markedly suppressed tumor growth in vivo without inducing evident systemic toxicity. Micro-PET/CT scans revealed a notable reduction in tumor ^18^F-FDG and ^18^F-FLT uptake in the DC-5163 treatment group compared to the DMSO control group.

**Conclusions:**

Our results suggest that DC-5163 is a promising GAPDH inhibitor for suppressing breast cancer growth without obvious side effects. ^18^F-FDG and ^18^F-FLT PET/CT can noninvasively assess the levels of glycolysis and proliferation in tumors following treatment with DC-5163.

## Background

Breast cancer poses a significant threat to women’s health, characterized by high morbidity and mortality rates. According to the World Health Organization (WHO), there were 2.3 million reported cases of breast cancer worldwide, resulting in 670,000 fatalities in 2022. Chemotherapy remains one of the most effective methods to treat breast cancer. However, most antitumor chemotherapeutic agents can diffuse and penetrate almost all organs to simultaneously damage tumor cells and normal healthy cells [1]. Such adverse effects pose long-term challenges for patients and are one of the major obstacles faced by oncologists. Therefore, more effective and safe treatments for cancer are needed.

Cancers exhibit aberrant metabolism characterized by aerobic glycolysis, and high glycolysis even in aerobic conditions and in the presence of unimpaired mitochondrial integrity [2, 3]. The difference in the regulation of glycolysis of normal cells and cancer cells provides targets for cancer therapy and drug discovery. Targeting glucose metabolism has been an attractive cancer treatment strategy, and several preclinical studies have proven the effectiveness of this therapeutic approach [4, 5].

Glyceraldehyde-3-phosphate dehydrogenase (GAPDH) is a key enzyme in glycolysis, and studies have suggested that it could serve as the rate-controlling enzyme in this metabolic pathway [6, 7]. Consequently, it has emerged as one of the most promising targets for antiglycolytic cancer therapy [8]. Up-regulated GAPDH is observed in multiple cancers and has been demonstrated to promote cancer cell survival, growth, and metastases [9]. Several GAPDH inhibitors, such as 3-bromopyruvate, koningic acid, and iodoacetate, have been studied preclinically in vitro and in vivo and have shown significant anticancer efficacy [10–12]. However, because of the systemic toxicity of these GAPDH inhibitors, their application is greatly limited. Therefore, the discovery of GADPH inhibitors with low cytotoxicity is of significance for cancer prevention and therapy.

In our previous study, we identified a new GAPDH inhibitor, DC-5163, using docking-based virtual screening and in vitro biochemical analysis [13]. DC-5163 inhibits the activity of GAPDH in various cancer cell lines and exerts anti-proliferation effects on cancer cells. However, the mechanism of the antiproliferative effect of DC-5163 and its antitumor activity and safety in vivo is still unknown.

The high rate of aerobic glycolysis is one of the prominent features of cancer cells. Therefore, ^18^F-fluorodeoxyglucose (^18^F-FDG) positron emission tomography/computed tomography (PET/CT) has been widely used in cancer diagnosis, including in clinical staging and efficacy monitoring [14, 15]. High levels of glycolysis allow cancer cells to sustain rapid proliferation and evade programmed cell death. Several antitumor drugs exert their functions by inhibiting cell proliferation and triggering apoptosis. Therefore, noninvasive imaging tools can be used to evaluate the changes in tumor proliferation and apoptosis in response to these drugs for early efficacy monitoring [16, 17]. The thymidine analog 3′-deoxy-3′-^18^F-fluorothymidine (^18^F-FLT) is phosphorylated by thymidine kinase 1 (TK1) and retained within proliferative cells; therefore, it can be used as a proliferation tracer. Several preclinical and clinical studies have confirmed the successful use of ^18^F-FLT to monitor tumor response to therapy [18]. The small molecule probe 2-(5-^18^ F-fluoropentyl)-2-methylmalonic acid (^18^F-ML-10) is incorporated by apoptotic cells and accumulates in cells, thus reflecting pathological changes with in vivo imaging [19, 20]. ^18^F-ML-10 is the first PET radiotracer for apoptosis imaging; it has entered the clinical stage and has a promising application prospect [21, 22].

In this study, we investigated the effects of DC-5163, a novel small-molecule inhibitor that targets GAPDH, on breast cancer in detail. We demonstrate that DC-5163 suppresses aerobic glycolysis activity in breast cancer cells, inhibits cell proliferation, and induces apoptosis in vitro and in vivo. Micro-PET/CT was shown to be effective for noninvasive evaluation during DC-5163 treatment in a mouse xenograft model. We also assessed the safety of DC-5163 by monitoring body weight alterations and conducting pathological examination of key organs.

## Methods

### Compound

DC-5163 was synthesized by MedChemExpress (Shanghai, China) and had a purity greater than or equal to 95%.

### Cell lines

The MDA-MB-231, MCF-7, and BT549 breast cancer cell lines were purchased from the Cell Bank of the Chinese Academy of Sciences (Shanghai, China). All cell lines were authenticated by STR and tested for mycoplasma contamination. MDA-MB-231 cells and MCF7 cells were cultured in DMEM containing 4.5 g/L glucose (Gibco, NY, USA), 10% fetal bovine serum (Gibco), and 1% penicillin/streptomycin (Gibco). BT549 cells were cultured in RPMI-1640 medium (Gibco) containing 10% fetal bovine serum, 1% penicillin/streptomycin, and 10 µg/mL insulin (Beyotime, Shanghai, China). All cells were cultured at 37 °C in 5% CO_2_.

### Glucose uptake, lactate production, and cellular ATP content assays

Cells were seeded in 24-well plates at a density of 5 × 10^4^ cells per well and cultured overnight. After attachment, cells were treated with 80 µM DC-5163 or DMSO for 24 h. The concentrations of glucose and lactate in the medium were measured by a glucose assay kit (Sigma, St. Louis, MO, USA) and a lactate assay kit (Solarbio, Beijing, China), respectively, following the manufacturer’s protocol. The cellular ATP levels were measured using a luminescence ATP assay kit (Beyotime). Data were normalized by cell numbers or protein concentrations.

### Cell uptake assays

Cells were seeded in 6-well plates and cultured overnight. Next, 80 µM DC-5163 or DMSO was added to each well and the culture was continued. After 24 h incubation, 10 µCi ^18^F-FDG (or ^18^F-FLT and ^18^F-ML-10) was added to the medium, and cells were cultured for 1 h. Radioactivity in cells and supernatants was measured by a gamma counter following a previously described method [23] and then cells were counted with a cell counter. In vitro ^18^F-FDG (or ^18^F-FLT and ^18^F-ML-10) uptake was calculated using the following formula: uptake %ID/10^5^= [B / (B + F) / cell number 10^5^] × 100%, where B indicates radioactivity in cells and F indicates radioactivity in supernatants.

### Glycolytic activity assay

The Agilent Seahorse XF96 Glycolytic Rate Assay (Agilent, Santa Clara, CA, USA) was used to evaluate the glycolytic activity. Cells were plated at a density of 3–5 × 10^3^ cells per well and cultured overnight. Cells were treated with 80 µM DC-5163 or DMSO for 24 h. The extracellular acidification rate (ECAR) was measured following the manufacturer’s protocol, and both basal and compensatory glycolytic proton efflux rates (glycoPER) were determined using the accompanying software (Agilent). All data were normalized by the protein concentrations in each well at the end of the assay.

### Cell counting 8 (CCK8) assay

The cytotoxicity of DC-5163 on breast cancer cells was investigated using a CCK8 kit (Beyotime) following the manufacturer’s instructions. Briefly, cells were seeded in 96-well plates at a density of 1 × 10^4^ cells per well and cultured overnight. Cells were then treated with different concentrations of DC-5163 for 24 h, 48 h, or 72 h. Each concentration was analyzed in triplicate wells and the experiment was repeated independently three times.

### Colony formation assay

MDA-MB-231, MCF-7, and BT549 cells were plated in 6-well plates at a density of 5–7 × 10^2^ cells per well and cultured overnight. Cells were then cultured in different concentrations of DC-5163. After treatment for 8 h, the medium was replaced with fresh complete medium, and cells were cultured for 7–10 days, during which the medium was replaced every 3–4 days. The cell colonies were fixed with 4% paraformaldehyde (Beyotime) and stained with a Crystal Violet Staining Solution (Beyotime).

### Apoptosis assay

The Annexin V–FITC Apoptosis Detection kit (Sigma) was used following the manufacturer’s instructions. Briefly, cells were plated in 12-well plates at a density of 1 × 10^5^ cells per well and cultured overnight. Cells were then treated with different concentrations of DC-5163 for 48 h. Cells were collected and stained with Annexin V-FITC for 15 min at room temperature, and propidine iodide (PI) was added to the cell suspension. The cells were analyzed using a flow cytometer (Beckman Cytoflex S).

### Cell cycle analysis

MCF-7, MDA-MB-231, and BT549 cells were seeded in 6-well plates at a density of 1 × 10^6^ cells per well and cultured overnight. Cells were treated with different concentrations of DC-5163 for 24 h. Cells were then collected, washed with ice-cold PBS, and stained with PI staining buffer in the dark for 30 min at room temperature. The cells were analyzed using a flow cytometer (Beckman Cytoflex S) and the data were analyzed using the ModFit LT 5.0 program.

### Western blot analysis

Western blot analysis was conducted following standard protocols. Cells were lysed in RIPA buffer (Sigma) and protein concentrations were quantified using a BCA Protein Assay Kit (Epizyme, Shanghai, China). Equal amounts of protein (30 µg) were separated by SDS-PAGE and transferred to PVDF membranes (Beyotime). The membranes were blocked in protein-free rapid blocking buffer (Epizyme) for 20 min at room temperature and incubated overnight with primary antibodies at 4 °C. After three washes in TBST (10 min each), the membranes were incubated with HRP-linked secondary antibody (Beyotime) for 1 h at room temperature. Protein signals were visualized using the ChemiDoc XRS system (BioRad) and quantified using densitometry. The primary antibodies used in this study were as follows: β-Actin antibody (Proteintech, Wuhan, China), GLUT1 antibody (Proteintech), β-tubulin antibody (Epizyme), cyclin D1 antibody (Zen-Bio, Chengdu, China), CDK4 antibody (Zen-Bio), TK1 antibody (Zen-Bio), and PARP antibody (Cell Signaling Technology, USA).

### Xenograft model and in vivo experiments

All animal experiments were approved by the Animal Experimental Ethics Committee of Ruijin Hospital, Shanghai Jiao Tong University School of Medicine, and performed following the guidelines of the National Institutes of Health Guide for the Care and Use of Laboratory Animals (Eighth Edition).

To establish breast cancer xenograft models, MDA-MB-231 cells were subcutaneously injected into the right flank of five-week-old immunodeficient female BALB/c nude mice (Lingchang Animal Experiment Center, Shanghai, China). When tumors reached approximately 50 mm^3^, mice were randomized to the DMSO control group and the DC-5163 treatment group (*n* = 6 per group). Mice in the DC-5163 group received intraperitoneal injections of DC-5163 (formulated in DMSO/PEG300/saline (5/25/70, v/v/v)) at a dosage of 80 mg/kg every two days, whereas mice in the DMSO control group were administered the vehicle solution only. The total injection volume was approximately 200 µL, with slight variations depending on the weight of each mouse. The weight and the volume of tumors were monitored every two days. The tumor volumes were calculated using the following formula: tumor volume (mm^3^) = width^2^ × length/2. At the endpoint, the mice were sacrificed, the blood was collected, and plasma was obtained by centrifugation. The tumors and key organs (heart, liver, spleen, lung, kidney) were harvested for further analysis.

### Micro-PET/CT imaging

Before and after treatment, a PET/CT scan was performed on tumor-bearing mice using an Inveon small-animal PET/CT system (Siemens, Munich, Germany). Mice were anesthetized under 2% isoflurane anesthesia and injected with 150–200 µCi ^18^F-FDG (or ^18^F-FLT) probe via the tail vein. After 45–60 min, animals were imaged with PET/CT under anesthesia (a 5-min CT scan and a 10-min PET scan). In particular, for the ^18^F-FDG study, the mice were kept fasting for 8 h before scans and remained under anesthesia state throughout injection, metabolism, and scanning.

### Image analysis

The images were reconstructed on an Inveon Acquisition Workplace. For data analysis, the region of interest (ROI) was manually delineated to encompass the entire tumor on CT images and then overlaid onto PET images. Subsequently, the uptake of ^18^F-FDG and ^18^F-FLT within the tumor ROIs was automatically analyzed and expressed as the percentage of injected dose per gram (%ID/g). The %ID/g was calculated by dividing the mean radioactivity of the ROI by the injected dose of radioactivity per kilogram of body weight.

### Immunohistochemistry (IHC)

Immunohistochemistry was performed following standard protocols. Briefly, tumors and organs were fixed in 4% paraformaldehyde (Beyotime) for 24 h, followed by standard tissue processing and embedding. Paraffin-embedded tissues were sectioned into 3–5 μm thick slices. Specimens were rehydrated with a concentration gradient of ethanol and distilled water. Conventional hematoxylin and eosin (H&E) staining was performed. Slices were incubated with an antibody against Ki67 (Servicebio, Wuhan, China) or GLUT1 (Proteintech) at 4 °C overnight, washed with PBS, and incubated with a secondary antibody at room temperature. Visualization was performed using a peroxidase substrate DAB kit.

### TUNEL assay

TUNEL assay was performed using a commercially available TUNEL kit (Servicebio) following the manufacturer’s instructions. Briefly, tissue slides were deparaffinized, and tumor specimens were treated with proteinase K, followed by saturation in 3% hydrogen peroxide for 10 min. Subsequently, the terminal deoxynucleotidyl transferase (TdT) reaction was performed, and the peroxidase binding site was visualized using DAB. The slides were then counterstained with hematoxylin, dehydrated, cleared, and mounted before examination under a microscope to detect apoptosis in the tumor tissues.

### Statistical analysis

Statistical analyses were carried out using GraphPad Prism version 9.0. All data are presented as mean ± SD. The differences between groups were analyzed by one-way analysis of variance (ANOVA) test and two-sided unpaired Student’s t-test. *P* < 0.05 was considered statistically significant. All experiments were performed at least three times, and the data were normalized by cell numbers or protein concentrations.

## Results

### DC-5163 suppresses aerobic glycolysis in breast cancer cells

**Fig. 1:**
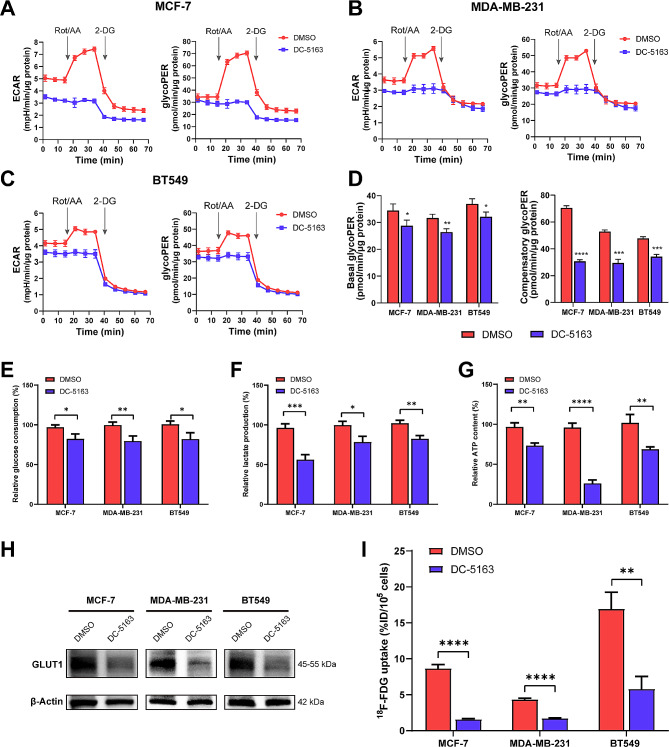
DC-5163 suppresses aerobic glycolysis in breast cancer cells. MCF-7, MDA-MB-231, and BT549 cells were treated with 80 µM DC-5163 or DMSO for 24 h. (**A–C**) The glycolytic activity was evaluated by Seahorse XF Glycolytic Rate Assay, ECAR, and glycoPER were measured with the administration of mitochondrial inhibitors Rot/AA and glycolytic inhibitor 2-DG. **D** Basal and compensatory glycoPER values were determined based on measurements from before and after administration of Rot/AA, respectively. Relative glucose consumption **E**, lactate production **F**, and cellular ATP content **G** were measured and calculated after treatment. **H** The protein level of GLUT1 was measured by Western blot analysis. **I** Cellular uptake of ^18^F-FDG was detected and calculated. Data are shown as mean ± SD (*n* = 3); * *P* < 0.05, ** *P* < 0.01, *** *P* < 0.001, and **** *P* < 0.0001 vs. the DMSO group

To evaluate whether glycolytic activity was suppressed following treatment with DC-5163, an inhibitor of GADPH, we used the Seahorse XF Glycolytic Rate Assay to evaluate the effects of DC-5163 in three breast cancer cell lines. As shown in Fig. 1A–C, both ECAR and glycoPER sharply increased after rotenone and antimycin A administration in DMSO-treated groups, suggesting ongoing compensatory glycolysis after mitochondrial inhibition. However, this effect was not observed in cells treated with DC-5163, indicating that intracellular glycolysis was inhibited. DC-5163 treatment strikingly reduced both the basal and compensatory glycoPER values in MCF-7, MDA-MB-231, and BT549 cells (Fig. 1D).

To further evaluate the effects of DC-5163 on the glycolysis of breast cancer cells, we then examined glucose consumption and lactate production. The results indicated that DC-5163 significantly inhibited glucose uptake (MCF-7, *P* < 0.05; MDA-MB-231, *P* < 0.01; BT549, *P* < 0.05) (Fig. 1E) and lactate production (MCF-7, *P* < 0.001; MDA-MB-231, *P* < 0.05; BT549, *P* < 0.01) (Fig. 1F) in the three breast cancer cell lines. The production of ATP, the main product of energy metabolism, was also inhibited by DC-5163 (Fig. 1G). The protein level of GLUT1 decreased in response to treatment with DC-5163 in breast cancer cells (Fig. 1H). As shown in Fig. 1I, DC-5163 also reduced the cellular ^18^F-FDG uptake compared with results in the control group. These data suggest that DC-5163 blocks the glycolytic pathway and suppresses aerobic glycolysis in breast cancer cells.

### DC-5163 inhibits breast cancer cell growth and induces cell cycle arrest

**Fig. 2:**
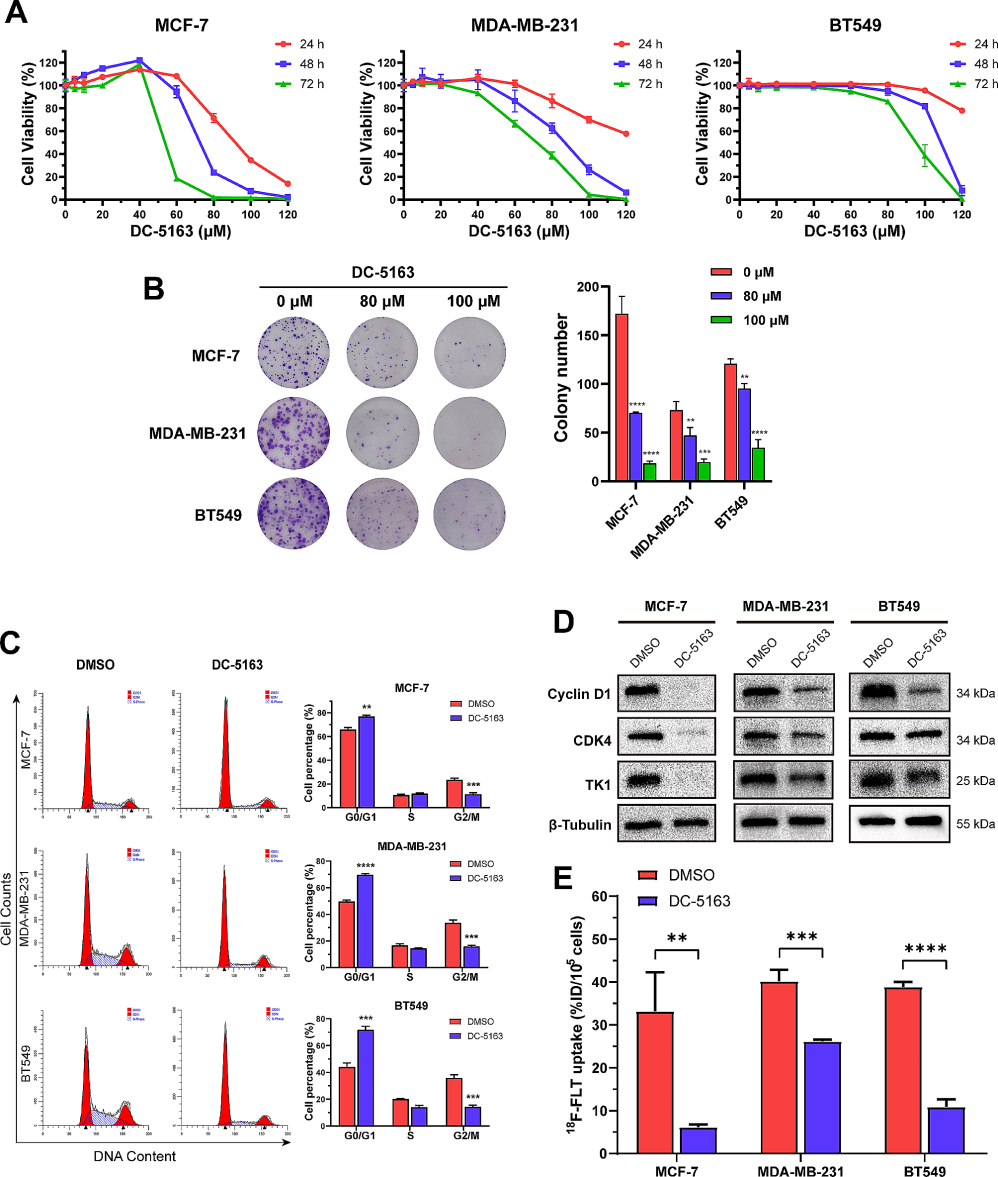
DC-5163 inhibits breast cancer cell growth and induces cell cycle arrest. MCF-7, MDA-MB-231, and BT549 cells were treated with different concentrations of DC-5163 for the indicated times. **A** Cell viability was assessed using the CCK8 assay. **B** Colony formation assay. **C** Cell cycle distribution was detected by flow cytometry. **D** The protein levels of cyclin D1, CDK4, and TK1 were measured by Western blot analysis. **E** Cellular uptake of ^18^F-FLT was detected and calculated. Data are shown as mean ± SD (*n* = 3); * *P* < 0.05, ** *P* < 0.01, *** *P* < 0.001, and **** *P* < 0.0001 vs. the DMSO group

We next examined the antineoplastic activity of DC-5163 in MCF-7, MDA-MB-231, and BT549 breast cancer lines using CCK8 assays. Cells were treated with various concentrations of DC-5163 for 24 h, 48 h, or 72 h. The results showed that DC-5163 inhibited cell viability in a dose- and time-dependent manner (Fig. 2A). We also examined the effect of DC-5163 on the colony formation capacity of the three breast cancer cell lines. DC-5163 markedly inhibited colony formation compared with the control, and the inhibition was also dose-dependent (Fig. 2B).

To further determine how DC-5163 affects cell proliferation, we analyzed the cell cycle distribution using flow cytometry. The results showed that DC-5163 induced cell cycle arrest at the G0/G1 phase (Fig. 2C). We further examined the protein expression of key regulators of the G1/S checkpoint by western blot assay and found that the expressions of cyclin D1 and CDK4 proteins were markedly decreased after DC-5163 treatment. The expression of the abnormal cell proliferation marker TK1 was also decreased (Fig. 2D). The retention of ^18^F-FLT in cells relies on TK1, and DC-5163 treatment significantly reduced the cellular ^18^F-FLT uptake (MCF-7, *P* < 0.01; MDA-MB-231, *P* < 0.001; BT549, *P* < 0.0001) (Fig. 2E).

Together, these findings indicate that DC-5163 exerts an effective and strong ability to inhibit cell proliferation and colony formation and induce G0/G1 phase arrest in breast cancer cells.

### DC-5163 induces apoptosis in breast cancer cells

**Fig. 3:**
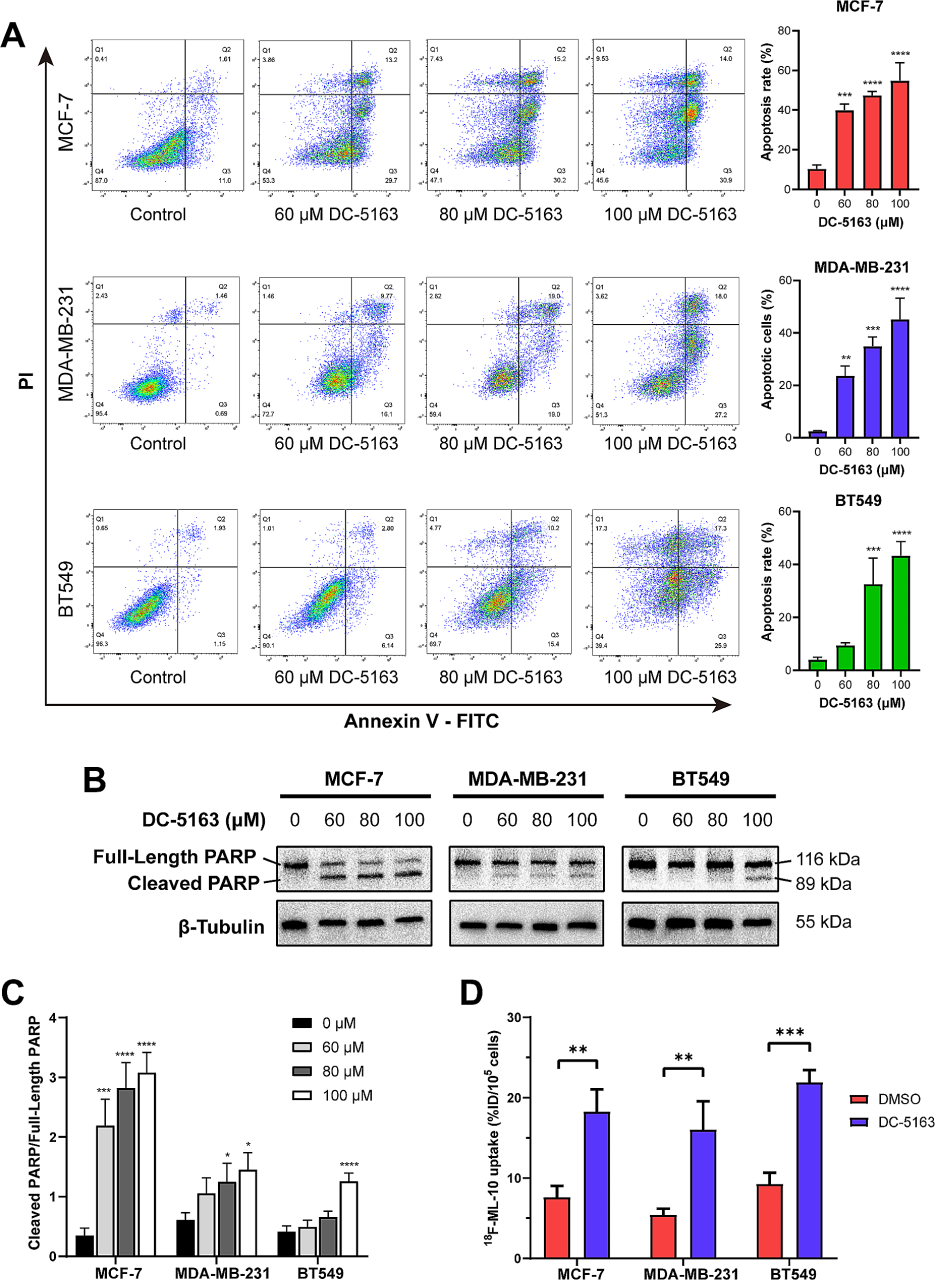
DC-5163 induces apoptosis in breast cancer cells. MCF-7, MDA-MB-231, and BT549 cells were treated with different concentrations of DC-5163 for 48 h. **A** The apoptosis rates were detected by flow cytometry. **B–C** The protein level of cleaved PARP was measured by Western blot analysis. **D** Cellular uptake of ^18^F-ML-10 was detected and calculated. Data are shown as mean ± SD (*n* = 3); * *P* < 0.05, ** *P* < 0.01, *** *P* < 0.001, and **** *P* < 0.0001 vs. the DMSO group

Annexin V–FITC/PI double staining assay was performed to demonstrate whether the antineoplastic activity of DC-5163 was from the induction of apoptosis. As shown in Fig. 3A, various concentrations of DC-5163 increased the percentage of cells in both early and late apoptosis in the three breast cancer cell lines compared with controls. Western blot assay showed that the expression of cleaved PARP, a marker of apoptosis, was significantly elevated after DC-5163 treatment (Fig. 3B–C). These findings confirmed the strong apoptotic-inducing effect of DC-5163.

As ^18^F-ML-10 can accumulate in apoptotic cells, we next conducted cell uptake experiments of ^18^F-ML-10 in vitro. The cellular ^18^F-ML-10 uptake was higher in the DC-5163 treatment group than in the control group (Fig. 3D). Collectively, these observations indicated that DC-5163 induces apoptosis in breast cancer cells.

#### In vivo antitumor activity and safety evaluation of DC-5163

**Fig. 4:**
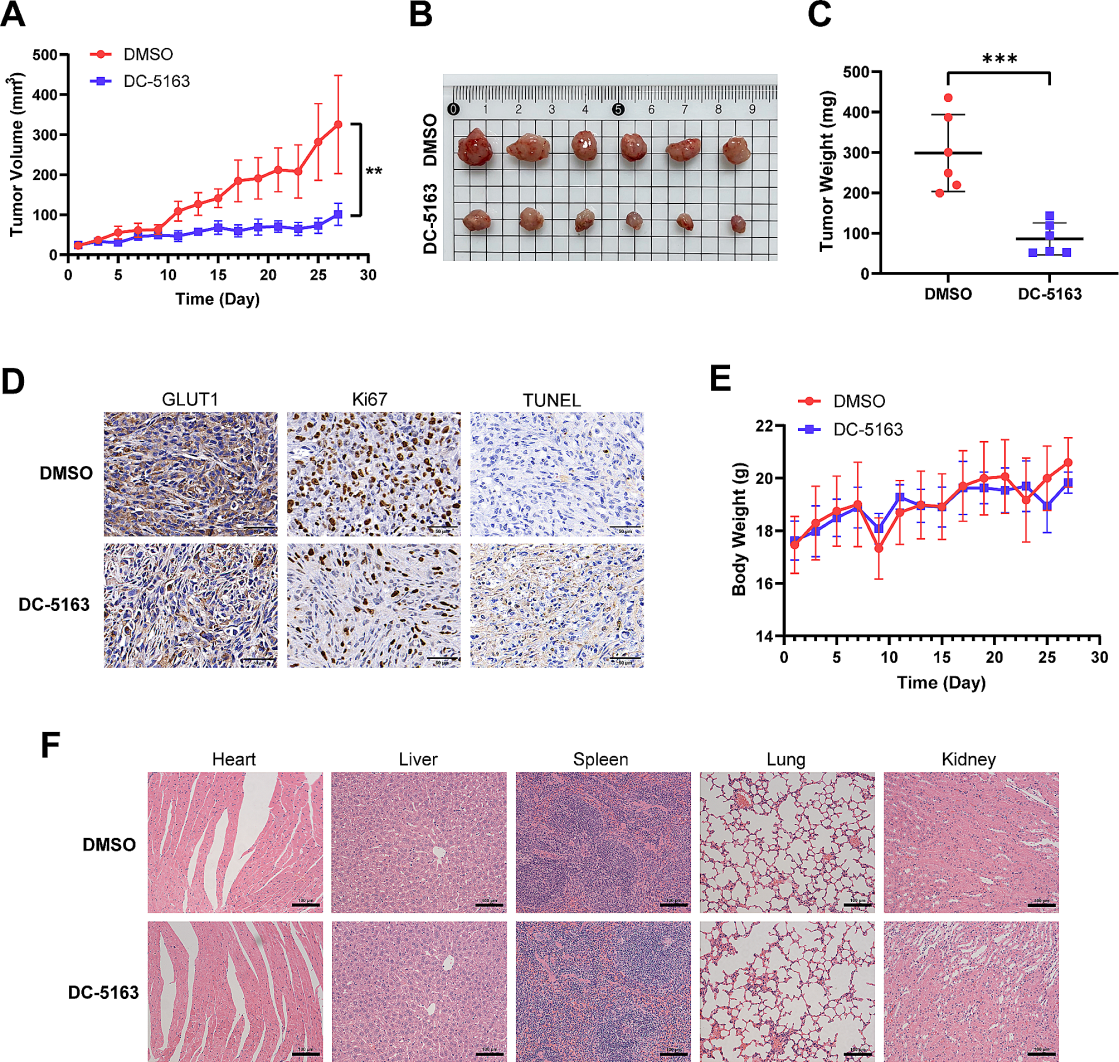
DC-5163 suppresses breast cancer in vivo with low cytotoxicity. The tumor-bearing mice were intraperitoneally injected with DC-5163 (80 mg/kg, q2d) or DMSO diluted with saline for 28 days. **A** The tumor volume growth curve was calculated to investigate tumorigenesis. **B** Photographs of tumor samples. **C** The weights of tumor samples. **D** Representative images of immunohistochemistry and TUNEL assay of the tumor samples (magnification, ×400). **E** Body weight of tumor-bearing mice. No significant difference in body weight was observed between the DC-5163 group and the control group. **F** HE staining images of vital organs (magnification, ×200). Data are shown as mean ± SD (*n* = 6); * *P* < 0.05, ** *P* < 0.01, *** *P* < 0.001, and **** *P* < 0.0001 vs. the DMSO group

We next evaluated the effect of DC-5163 treatment in vivo using a xenograft mouse model. MDA-MB-231 cells belong to triple-negative breast cancer with a high malignancy degree, and experiments in vitro showed that DC-5163 had acceptable IC_50_ against MDA-MB-231 cells. Therefore, MDA-MB-231 was selected to establish the xenograft model. As shown in Fig. 4A, the tumor volume of the DC-5163 group decreased during the treatment compared with controls. At the endpoint, both the tumor volume and weight of the DC-5163 treatment group were significantly reduced compared with those of the control group (volume, *P* < 0.01; weight, *P* < 0.001) (Fig. 4B–C), indicating that DC-5163 had strong antitumor activity in vivo.

The immunohistochemistry assay revealed a decrease in GLUT1 expression in the DC-5163 treatment group compared to the DMSO group. Additionally, the expression of Ki67, a crucial marker for cancer proliferation, was also reduced in the DC-5163 group. TUNEL assay showed that the apoptotic cell population in the DC-5163 group was increased (Fig. 4D). These results further indicated that DC-5163 suppresses breast cancer by blocking the glycolytic pathway, inhibiting proliferation and inducing apoptosis.

We next considered the therapeutic safety of DC-5163. There was no significant difference in body weight between the DC-5163 group and the control group during the treatment (*P* > 0.05, Fig. 4E). No significant morphological changes were observed in the heart, liver, spleen, lung, and kidney tissue in the DC-5163 treatment group (Fig. 4F), indicating low cytotoxicity of DC-5163 in vivo. To further confirm the low toxicity of DC-5163, we collected blood samples from mice and tested hepatic and renal functions as well as hematological parameters. As shown in Tables 1 and 80 mg/kg DC-5163 had no hematotoxicity in the peripheral blood of mice and no significant effect on hematological indexes (*P* > 0.05). Additionally, there were no significant differences in hepatic function parameters and renal function parameters in the DC-5163 group, indicating that DC-5163 had no significant hepatorenal toxicity. These observations suggest that DC-5163 may inhibit tumor growth in vivo without significant side effects.

**Table 1:** Comparison of hematological parameters

Hematological parameters	DMSO	DC-5163	*P* values
ALT(U/L)	39.44 ± 4.440	39.03 ± 2.592	0.8477
AST(U/L)	93.65 ± 16.79	92.92 ± 19.85	0.9464
BUN(mg/dL)	21.17 ± 1.065	22.21 ± 2.551	0.3752
CR(µmol/L)	54.50 ± 5.882	48.15 ± 12.50	0.2861
UA(µmol/L)	90.21 ± 7.991	86.17 ± 13.98	0.5527
WBC(10^9/L)	4.233 ± 1.429	3.150 ± 1.529	0.2336
RBC(10^12/L)	9.177 ± 0.2781	8.848 ± 0.4881	0.1828
Hb(g/L)	134.2 ± 6.242	129.2 ± 11.29	0.3647

**ALT**: Alanine transaminase; **AST**: Aspartate aminotransferase; **BUN**: Blood urea nitrogen; **CR**: Creatinine; **UA**: Uric acid; **WBC**: White blood cell; **RBC**: Red blood cell; **Hb**: Hemoglobin

### Monitoring of the efficacy of DC-5163 by molecular imaging

**Fig. 5:**
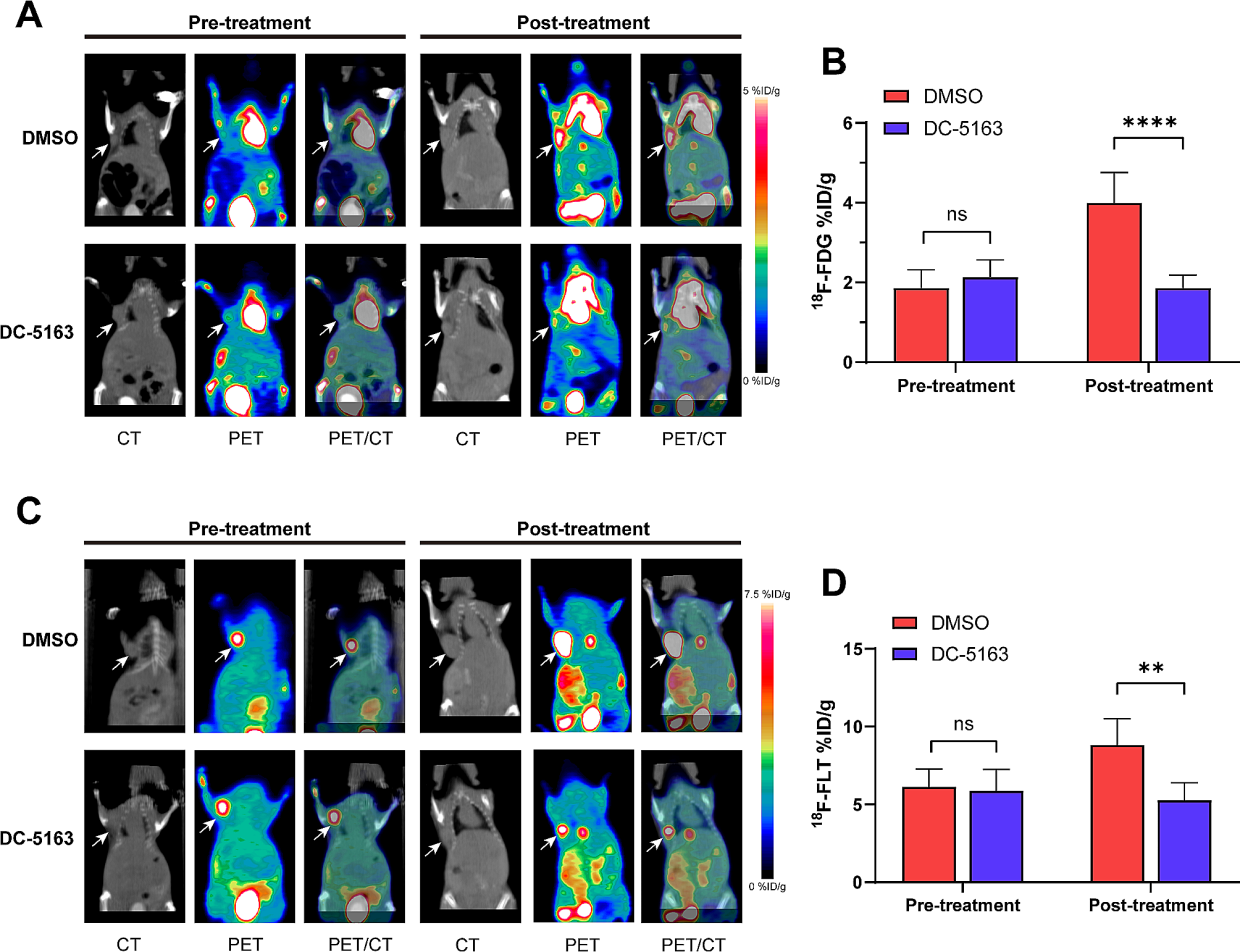
Monitoring of the efficacy of DC-5163 in vivo by molecular imaging. The tumor-bearing mice underwent micro-PET/CT scans before and after the treatment of DC-5163 or DMSO. **A** Representative images acquired by ^18^F-FDG micro-PET/CT scanning. **B** Tumor uptake of ^18^F-FDG was measured by VOI analysis in mice bearing MDA-MB-231 human breast cancer xenografts. **C** Representative images acquired by ^18^F-FLT micro-PET/CT scanning. **D** Tumor uptake of ^18^F-FLT was measured by VOI analysis in tumor-bearing mice. Data are presented as average %ID/g (mean ± SD, *n* = 6); ns, no significance, * *P* < 0.05, ** *P* < 0.01, *** *P* < 0.001, and **** *P* < 0.0001 vs. the DMSO group

To monitor the therapeutic efficacy of DC-5163 in vivo, micro-PET/CT scanning was performed. Mirroring the trend observed in the tumor volume growth curve, levels of glucose metabolism and proliferation in the tumors of DMSO control mice markedly increased from baseline as tumors progressed. Regions of interest (ROIs) were delineated, and the percentage injected dose per gram (%ID/g) was obtained for quantitative analysis. No significant differences between the two groups were observed before treatment. Consistent with the results of uptake assays in vitro, mice treated with DC-5163 exhibited a striking decrease in tumor ^18^F-FDG and ^18^F-FLT uptake. After 4 weeks of treatment, the %ID/g values in the DC-5163 group decreased significantly compared to the DMSO group, indicating a decrease in glucose metabolism and proliferation levels (^18^F-FDG, *P* < 0.0001;^18^F-FLT, *P* < 0.01) (Fig. 5A–D). Furthermore, representative micro-PET/CT images showed that the tumor sizes in the DC-5163 treatment group were smaller than those in the DMSO group, providing further evidence of the antitumor activity of DC-5163.

## Discussion

To meet the increased energy metabolic demands for cancer development, the rate of glycolysis in cancer cells is increased to nearly ten times that of normal cells [24, 25]. One reason for the increased rate of glycolysis is the overexpression of specific glycolytic enzymes [26]. These findings suggest that inhibiting the expression or activity of glycolytic enzymes may be a potential approach to suppress cancer cells. GAPDH is a classical glycolytic enzyme that also acts as a moonlighting protein and is involved in tumor progression, invasiveness, and metastases [9]. The identification of effective GAPDH inhibitors with clinical translational value is critical for developing new therapeutic methods for cancer treatment [27, 28].

We previously reported DC-5163 as a new small-molecule inhibitor of GAPDH. DC-5163 inhibited GAPDH activity in five different cancer cell lines including human breast cancer cells, human colon cancer cells, and human lung cancer cells. Furthermore, DC-5163 significantly inhibited the growth of MDA-MB-231 breast cancer cells but had no effect on MCF-10 A normal breast epithelial cells [13]. These findings suggest that DC-5163 may be a promising candidate drug for breast cancer therapy.

In this study, we further explored the mechanism and effects of DC-5163 in breast cancer cells and its efficacy in vivo. First, we found that DC-5163 treatment remarkably decreased the ECAR, glycoPER, glucose uptake, ^18^F-FDG uptake, lactate, and ATP production in breast cancer cells. These findings indicate that DC-5163 inhibits GAPDH and blocks the glycolytic pathway. We then found that DC-5163 significantly inhibited proliferation and clone formation ability and induced cell cycle arrest in the G0/G1 phase in three breast cancer cell lines. Furthermore, the proportion of apoptotic cells increased significantly after treatment with DC-5163 in a dose-dependent manner. Of the three breast cancer cells, MCF-7 had a lower IC_50_ value, probably because it was less malignant and aggressive than the other two. We speculate that along with inhibition of glycolysis, breast cancer cells treated with DC-5163 experienced a rapid, extensive ATP deprivation, and reduced energy supply led to stalled proliferation and increased apoptosis. These changes were accompanied by the increased or decreased uptake of specific imaging agents and suggested the potential for molecular imaging to evaluate the efficacy of DC-5163 in tumor treatment.

One of the current challenges in oncology is to develop imaging tools for early detection of the response to chemotherapy and adjust treatment strategies when necessary [29]. As a representative “trans-pathological” approach, molecular imaging with PET enables safe and comprehensive evaluation of disease biological processes [30, 31]. Therefore, we established a xenograft mouse model and used micro-PET/CT to monitor the efficacy of DC-5163 in vivo. ^18^F-FDG PET/CT is the gold standard for PET imaging in breast cancer, especially for tumor staging, detection of recurrent disease, and monitoring of treatment response [32]. We performed ^18^F-FDG-micro-PET/CT scanning before and after the treatment of DC-5163. We found that DC-5163 significantly reduced tumor uptake of ^18^F-FDG (SUVmean and SUVmax) compared with the control. The uptake of ^18^F-FDG relies on the glucose consumption of tumor tissue [33], and decreased uptake was the result of the reduction in the overall glycolysis level of the tumor after DC-5163 treatment. However, some inflammatory cells also show elevated glucose metabolism levels, which can lead to false positives in ^18^F-FDG imaging. Thus, to analyze the biological response more accurately after DC-5163 treatment, new specific probes need to be developed.

Most antitumor drugs work by inhibiting proliferation and inducing apoptosis. Therefore, noninvasive imaging tools that can evaluate the changes in tumor proliferation and apoptosis after treatment can be used for early assessment of the therapeutic response. ^18^F-FLT is a proliferation tracer that has better discrimination between tumor and inflammatory tissue compared with ^18^F-FDG [34]. In this study, we performed ^18^F-FLT PET/CT scanning in the mouse model before and after treatment with DC-5163. We observed that both SUVmean and SUVmax of ^18^F-FLT were decreased markedly in the DC-5163 treatment group. For efficacy monitoring, uptake of ^18^F-FLT is largely determined by intracellular TK1 enzyme levels after drug administration [35]. Suzuki and colleagues reported that PD1 blockade increased the G2/M population in murine colon carcinoma cells, which have high TK1 activity. Therefore, ^18^F-FLT was taken up by tumor cells, even if tumor proliferation was suppressed [17]. However, in our study, DC-5163 induced cell cycle arrest at the G0/G1 phase, and TK1 activity was relatively low. We thus found that cancer growth inhibition after DC-5163 treatment coincides with reduced tumor uptake of ^18^F-FLT. These findings also show the importance of a deep understanding of cellular mechanisms for selecting optimal tracers for monitoring drug efficacy.

Apoptosis is the main type of chemotherapy-induced cell death. The emergence of new nuclear imaging techniques has made it possible to monitor apoptosis during treatment [36]. ^18^F-ML-10 is a radiotracer that was confirmed to specifically detect apoptotic cells and discriminate apoptotic cells from cells that died from other forms of cell death [37]. It has entered the clinical stage and exhibited promising results in some small-scale clinical practices [36]. However, conflicting findings have arisen from various research groups regarding the efficacy of ^18^F-ML-10 in monitoring treatment response, some studies indicate that increased apoptosis during treatment does not consistently correlate with the tumor uptake of ^18^F-ML-10 [29, 38, 39]. In our study, we utilized ^18^F-ML-10 to detect apoptotic cells, yielding results only in vitro. Despite observing increased apoptotic cells in tumor tissues after DC-5163 treatment via TUNEL staining, the obtained micro-PET/CT images exhibited poor quality and no significant uptake of ^18^F-ML-10 was discernible in the tumor ROIs. Therefore, the sole reliance on ^18^F-ML-10 for efficacy monitoring warrants further investigation, and its utility in the early evaluation of tumor chemotherapy treatments necessitates additional research.

## Conclusions

**Fig. 6:**
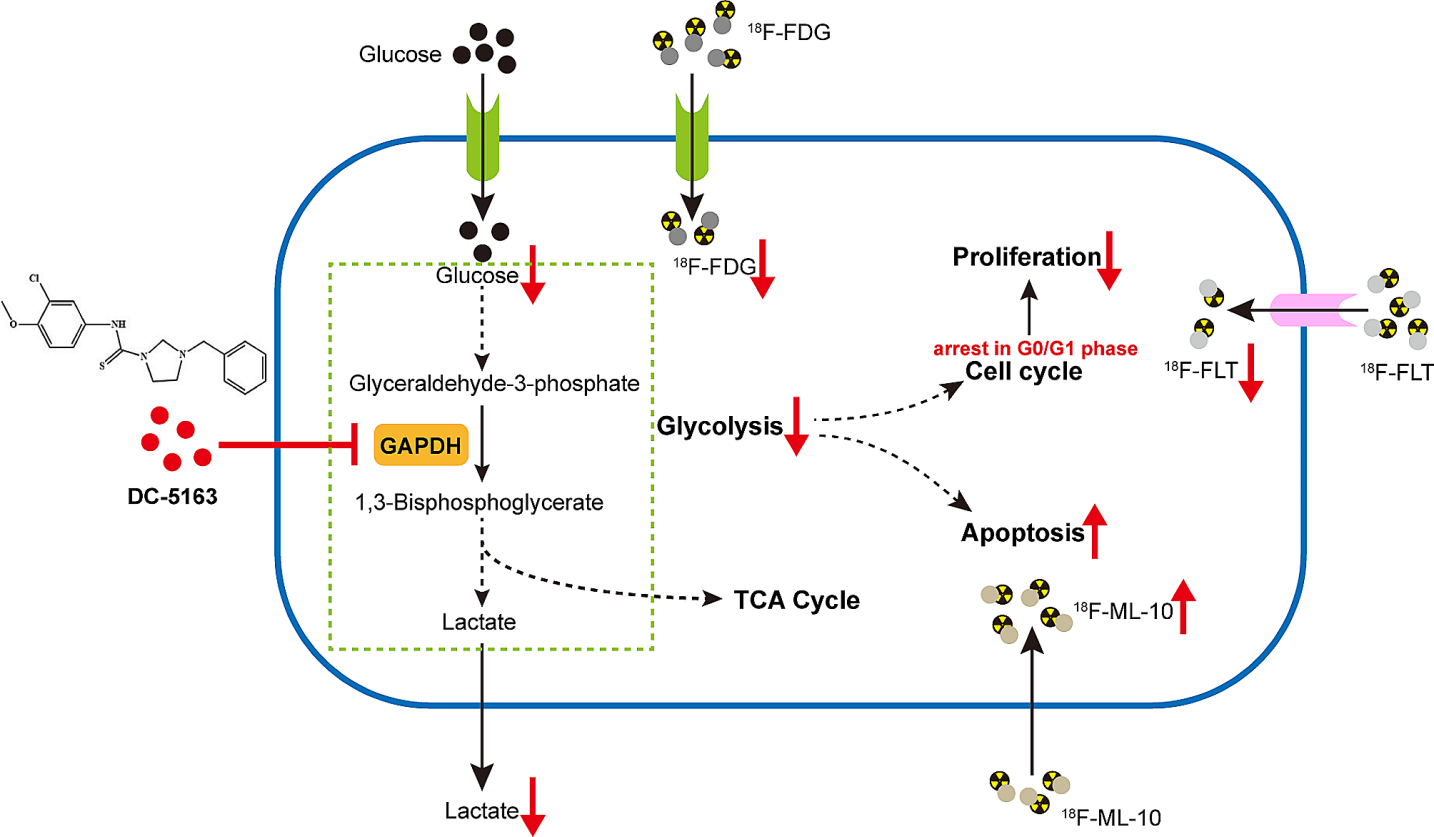
Proposed model of the mechanism underlying the anti-tumor activity of compound DC-5163. In addition to inhibition of glycolysis, breast cancer cells treated with DC-5163 undergo a rapid, extensive energy supply deprivation, leading to halted proliferation and increased apoptosis. These changes were accompanied by the increased or decreased uptake of specific imaging agents, making it possible to monitor treatment effects using PET/CT

Taken together, our results demonstrated that DC-5163, a novel inhibitor targeting GAPDH, suppressed aerobic glycolysis and reduced the energy supply of breast cancer cells, thereby leading to the inhibition of proliferation and increase of apoptosis. Furthermore, ^18^F-FDG and ^18^F-FLT micro-PET/CT were successfully used for noninvasive assessment of glucose metabolism and proliferation levels in mouse xenograft model during the treatment. Our findings not only provide novel insights into DC-5163 as a promising GAPDH inhibitor for suppressing breast cancer but also indicate that PET/CT may be used as an effective noninvasive method for monitoring the efficacy of DC-5163 treatment (Fig. 6).

## Data Availability

No datasets were generated or analysed during the current study.

## Abbreviations

GAPDH: Glyceraldehyde-3-phosphate dehydrogenase
PET/CT: Positron emission tomography/computed tomography
^18^F-FDG: 2′-deoxy-2′-^18^F-fluoro-D-glucose
^18^F-FLT: 3′-deoxy-3′-^18^F-fluorothymidine
^18^F-ML-10: 2-(5-^18^ F-fluoropentyl)-2-methylmalonic acid
ROI: Region of interest
%ID/g: The percentage of injected dose per gram

## References

[1] Jin S, Ye K (2013). Targeted drug delivery for breast cancer treatment. Recent Pat Anticancer Drug Discov.

[2] Warburg O (1956). On the origin of cancer cells. Science.

[3] Hanahan D, Weinberg RA (2011). Hallmarks of cancer: the next generation. Cell.

[4] Galluzzi L, Kepp O, Vander Heiden MG, Kroemer G (2013). Metabolic targets for cancer therapy. Nat Rev Drug Discov.

[5] Marchiq I, Pouysségur J (2016). Hypoxia, cancer metabolism and the therapeutic benefit of targeting lactate/H(+) symporters. J Mol Med (Berl).

[6] Liberti MV, Dai Z, Wardell SE, Baccile JA, Liu X, Gao X, Baldi R, Mehrmohamadi M, Johnson MO, Madhukar NS (2017). A predictive model for selective targeting of the Warburg Effect through GAPDH inhibition with a natural product. Cell Metab.

[7] Shestov AA, Liu X, Ser Z, Cluntun AA, Hung YP, Huang L, Kim D, Le A, Yellen G, Albeck JG et al. Quantitative determinants of aerobic glycolysis identify flux through the enzyme GAPDH as a limiting step. Elife 2014, 3. 10.7554/eLife.03342 PMC4118620 25009227

[8] Abdel-Wahab AF, Mahmoud W, Al-Harizy RM (2019). Targeting glucose metabolism to suppress cancer progression: prospective of anti-glycolytic cancer therapy. Pharmacol Res.

[9] Sirover MA (2018). Pleiotropic effects of moonlighting glyceraldehyde-3-phosphate dehydrogenase (GAPDH) in cancer progression, invasiveness, and metastases. Cancer Metastasis Rev.

[10] Pereira da Silva AP, El-Bacha T, Kyaw N, dos Santos RS, da-Silva WS, Almeida FC, Da Poian AT, Galina A (2009). Inhibition of energy-producing pathways of HepG2 cells by 3-bromopyruvate. Biochem J.

[11] Kumagai S, Narasaki R, Hasumi K (2008). Glucose-dependent active ATP depletion by koningic acid kills high-glycolytic cells. Biochem Biophys Res Commun.

[12] Reda A, Refaat A, Abd-Rabou AA, Mahmoud AM, Adel M, Sabet S, Ali SS (2019). Role of mitochondria in rescuing glycolytically inhibited subpopulation of triple negative but not hormone-responsive breast cancer cells. Sci Rep.

[13] Li T, Tan X, Yang R, Miao Y, Zhang M, Xi Y, Guo R, Zheng M, Li B (2020). Discovery of novel glyceraldehyde-3-phosphate dehydrogenase inhibitor via docking-based virtual screening. Bioorg Chem.

[14] Kelloff GJ, Hoffman JM, Johnson B, Scher HI, Siegel BA, Cheng EY, Cheson BD, O’Shaughnessy J, Guyton KZ, Mankoff DA (2005). Progress and promise of FDG-PET imaging for cancer patient management and oncologic drug development. Clin Cancer Res.

[15] Kitajima K, Yamano T, Fukushima K, Miyoshi Y, Hirota S, Kawanaka Y, Miya M, Doi H, Yamakado K, Hirota S (2016). Correlation of the SUVmax of FDG-PET and ADC values of diffusion-weighted MR imaging with pathologic prognostic factors in breast carcinoma. Eur J Radiol.

[16] Heinzmann K, Nguyen QD, Honess D, Smith DM, Stribbling S, Brickute D, Barnes C, Griffiths J, Aboagye E (2018). Depicting changes in Tumor Biology in response to Cetuximab Monotherapy or Combination Therapy by apoptosis and proliferation imaging using (18)F-ICMT-11 and (18)F-FLT PET. J Nucl Med.

[17] Suzuki M, Matsuda T, Nakajima K, Yokouchi Y, Kuge Y, Ogawa M (2022). PD1 blockade alters cell-cycle distribution and affects 3’-deoxy-3’-[(18)F]fluorothymidine uptake in a mouse CT26 tumor model. Ann Nucl Med.

[18] Schelhaas S, Wachsmuth L, Hermann S, Rieder N, Heller A, Heinzmann K, Honess DJ, Smith DM, Fricke IB, Just N (2018). Thymidine metabolism as a confounding factor for 3’-Deoxy-3’-(18)F-Fluorothymidine Uptake after Therapy in a Colorectal Cancer Model. J Nucl Med.

[19] Simpson KL, Cawthorne C, Zhou C, Hodgkinson CL, Walker MJ, Trapani F, Kadirvel M, Brown G, Dawson MJ, MacFarlane M (2013). A caspase-3 ‘death-switch’ in colorectal cancer cells for induced and synchronous tumor apoptosis in vitro and in vivo facilitates the development of minimally invasive cell death biomarkers. Cell Death Dis.

[20] Cohen A, Shirvan A, Levin G, Grimberg H, Reshef A, Ziv I (2009). From the gla domain to a novel small-molecule detector of apoptosis. Cell Res.

[21] Allen AM, Ben-Ami M, Reshef A, Steinmetz A, Kundel Y, Inbar E, Djaldetti R, Davidson T, Fenig E, Ziv I (2012). Assessment of response of brain metastases to radiotherapy by PET imaging of apoptosis with ¹^8^F-ML-10. Eur J Nucl Med Mol Imaging.

[22] Oborski MJ, Laymon CM, Lieberman FS, Drappatz J, Hamilton RL, Mountz JM (2014). First use of (18)F-labeled ML-10 PET to assess apoptosis change in a newly diagnosed glioblastoma multiforme patient before and early after therapy. Brain Behav.

[23] Xi Y, Li T, Xi Y, Zeng X, Miao Y, Guo R, Zhang M, Li B (2022). Combination treatment with hENT1 and miR-143 reverses gemcitabine resistance in triple-negative breast cancer. Cancer Cell Int.

[24] Li L, Liu H, Du L, Xi P, Wang Q, Li Y, Liu D (2018). miR-449a suppresses LDHA-Mediated glycolysis to enhance the sensitivity of Non-small Cell Lung Cancer cells to Ionizing Radiation. Oncol Res.

[25] Sun K, Hu P, Xu F (2018). LINC00152/miR-139-5p regulates gastric cancer cell aerobic glycolysis by targeting PRKAA1. Biomed Pharmacother.

[26] Dai S, Peng Y, Zhu Y, Xu D, Zhu F, Xu W, Chen Q, Zhu X, Liu T, Hou C (2020). Glycolysis promotes the progression of pancreatic cancer and reduces cancer cell sensitivity to gemcitabine. Biomed Pharmacother.

[27] Krasnov GS, Dmitriev AA, Snezhkina AV, Kudryavtseva AV (2013). Deregulation of glycolysis in cancer: glyceraldehyde-3-phosphate dehydrogenase as a therapeutic target. Expert Opin Ther Targets.

[28] Guo C, Liu S, Sun MZ (2013). Novel insight into the role of GAPDH playing in tumor. Clin Transl Oncol.

[29] Jouberton E, Schmitt S, Maisonial-Besset A, Chautard E, Penault-Llorca F, Cachin F (2021). Interest and limits of [(18)F]ML-10 PET imaging for early detection of response to Conventional Chemotherapy. Front Oncol.

[30] Zhao Q, He X, Qin X, Liu Y, Jiang H, Wang J, Wu S, Zhou R, Yu C, Liu S (2022). Enhanced therapeutic efficacy of combining Losartan and Chemo-Immunotherapy for Triple negative breast Cancer. Front Immunol.

[31] Tian M, He X, Jin C, He X, Wu S, Zhou R, Zhang X, Zhang K, Gu W, Wang J (2021). Transpathology: molecular imaging-based pathology. Eur J Nucl Med Mol Imaging.

[32] Groheux D, Cochet A, Humbert O, Alberini JL, Hindié E, Mankoff D (2016). ¹^8^F-FDG PET/CT for staging and restaging of breast Cancer. J Nucl Med.

[33] Miao Y, Zhang LF, Guo R, Liang S, Zhang M, Shi S, Shang-Guan CF, Liu MF, Li B (2016). (18)F-FDG PET/CT for monitoring the response of breast Cancer to mir-143-Based therapeutics by targeting Tumor Glycolysis. Mol Ther Nucleic Acids.

[34] Krys D, Hamann I, Wuest M, Wuest F (2019). Effect of hypoxia on human equilibrative nucleoside transporters hENT1 and hENT2 in breast cancer. Faseb j.

[35] Direcks WG, Berndsen SC, Proost N, Peters GJ, Balzarini J, Spreeuwenberg MD, Lammertsma AA, Molthoff CF (2008). [18F]FDG and [18F]FLT uptake in human breast cancer cells in relation to the effects of chemotherapy: an in vitro study. Br J Cancer.

[36] Qin X, Jiang H, Liu Y, Zhang H, Tian M (2022). Radionuclide imaging of apoptosis for clinical application. Eur J Nucl Med Mol Imaging.

[37] Wang X, Feng H, Zhao S, Xu J, Wu X, Cui J, Zhang Y, Qin Y, Liu Z, Gao T (2017). SPECT and PET radiopharmaceuticals for molecular imaging of apoptosis: from bench to clinic. Oncotarget.

[38] Jouberton E, Schmitt S, Chautard E, Maisonial-Besset A, Roy M, Radosevic-Robin N, Chezal JM, Miot-Noirault E, Bouvet Y, Cachin F (2020). [(18)F]ML-10 PET imaging fails to assess early response to neoadjuvant chemotherapy in a preclinical model of triple negative breast cancer. EJNMMI Res.

[39] Demirci E, Ahmed R, Ocak M, Latoche J, Radelet A, DeBlasio N, Mason NS, Anderson CJ, Mountz JM (2017). Preclinical evaluation of (18)F-ML-10 to determine timing of apoptotic response to Chemotherapy in Solid tumors. Mol Imaging.

